# Mutual effects of gestational diabetes and schizophrenia: how can one promote the other?: A review

**DOI:** 10.1097/MD.0000000000038677

**Published:** 2024-06-21

**Authors:** Kholoud A. Ghamri

**Affiliations:** a Department of Internal Medicine, King Abdulaziz University, Jeddah, Saudi Arabia.

**Keywords:** diabetes, gestational diabetes, mental health, psychiatric conditions, schizophrenia

## Abstract

Although the physical complications of gestational diabetes mellitus (GDM) are well known, emerging evidence suggests a significant link with psychiatric conditions such as schizophrenia (SCZ). This review aimed to explore the extent, nature, and implications of the association between GDM and SCZ, exploring how the 2 conditions may reciprocally influence each other. We conducted a comprehensive literature review and, analyzed clinical and mechanistic evidence supporting the mutual effects of GDM and SCZ. This review examined factors such as neurodevelopment and the impact of antipsychotics. The study found that Maternal GDM increases the risk of SCZ in offspring. Conversely, women with SCZ were more prone to hyperglycemic pregnancies. The research highlights significant regional variations in GDM prevalence, with the highest rate in the Middle East, North Africa, and South-East Asia regions. These regional variations may have an impact on the epidemiology of SCZ. Furthermore, this review identifies the potential biological and environmental mechanisms underlying these associations. There is a bidirectional relationship between GDM and SCZ, with each disorder potentially exacerbating the others. This relationship has significant implications for maternal and offspring health, particularly in regions with high GDM prevalence. These findings underline the need for integrated care approaches for women with SCZ during pregnancy and the importance of monitoring and managing GDM to mitigate the risk of SCZ in the offspring. Notably, this study recognizes the need for further research to fully understand these complex interactions and their implications for healthcare.

## 1. Introduction

Gestational diabetes mellitus (GDM) affects a considerable proportion of pregnancies, with varying prevalence globally. According to the International Diabetes Federation estimates, the global prevalence of GDM reaches 14.0%, with the highest rates reported in the Middle East and North Africa (27.6%) and South-East Asia (20.8%) countries.^[1]^ GDM exposes to multiple maternal complications such as pre-eclampsia, preterm labor, polyhydramnios, increased operative delivery, and infection.^[2]^ Additionally, fetal and neonatal complications include macrosomia, preterm birth, and metabolic disorders (hypoglycemia, hypocalcemia, hyperbilirubinemia), in addition to hematologic, respiratory, neurological, and digestive disorders.^[3]^ Therefore, the management of GDM is crucial for the health of both mother and fetus. Several risk factors have been shown to increase the likelihood of GDM, and schizophrenia (SCZ) is one of them.^[4]^ Conversely, epidemiological studies indicate that offspring born to mothers with GDM have a 7-fold increase in the risk of developing SCZ later in life compared to offspring from GDM-free pregnancies.^[5]^

SCZ is a complex neurodevelopmental disorder that predominantly affects individuals in late adolescence and early adulthood. It generally presents with cognitive and emotional impairments, which are often associated with positive symptoms such as hallucinations and delusions, and negative symptoms, including avolition (lack of motivation), alogia (reduced speech fluency), and apathy.^[6]^ This condition can severely impact health, social life, and productivity. It also poses a concern for both auto- and hetero-aggressive behaviors. The epidemiology of SCZ shows alarming trends. The Global Burden of Disease data reveal an increase in the global raw prevalence of SCZ from 14.2 million in 1990 to 23.6 million in 2019, representing a 65% rise. Similarly, the global incidence and disability-adjusted life years increased by 37% (941,000–1.3 million) and 65% (9.1–15.1 million), respectively, over the same period.^[7]^ This points towards the need for effective strategies to reduce the burden of SCZ globally, which requires a thorough understanding of the disease-related risk factors.

The current knowledge regarding the pathogenesis of SCZ remains incomplete. Novel hypotheses have emerged that involve biological insults in the neurodevelopmental process in early life. Similarly, the etiopathogenesis of GDM is not fully understood. Additionally, existing literature primarily focuses on the physical outcomes of GDM and SCZ separately, with little attention given to their mutual effects and possible overlapping mechanisms. In particular, there is a paucity of comprehensive reviews on the existing links between GDM and SCZ.

Considering this gap, exploring the association between GDM and SCZ could yield insightful outcomes. Therefore, we conducted a scoping review to elucidate the relationship between GDM and SCZ, both clinically and mechanistically. It aims to identify, map, and synthesize the existing literature on the established and hypothetical mutual effects of GDM and SCZ. This exploration could enhance our understanding of the etiologies of both diseases and guide future research aimed at developing preventive measures and public health strategies.

## 2. Method

We conducted an extensive search for publications relevant to GDM in the context of SCZ and vice versa across multiple databases including PubMed, Google Scholar, Scopus, and Web of Science. We utilized the following keywords, either individually or in various combinations: gestational diabetes, SCZ, hyperglycemia in utero, insulin resistance, neurodevelopment, neuroplasticity, neuroinflammation, the 2-hit hypothesis, antipsychotics, stress hormones, and the hypothalamic-pituitary-adrenal axis. Only the most insightful publications are included in this review. As this is a literature review, no ethical review or approval was necessary for this work.

## 3. Summary of the current clinical evidence

### 3.1. The risk of GDM in SCZ patients

A growing body of evidence indicates a higher risk of GDM among women with SCZ. A recent meta-analysis of observational studies, which included 518,592 women with 640,792 pregnancies, has shown that the pooled odds ratio (OR) of GDM at preconception is 2.44 among patients with SCZ, compared to those without SCZ.^[4]^ In a national register-based follow-up study on 1162 patients and 4683 controls, Simoila et al showed a significantly higher OR for pathologic oral glucose tolerance test (OR = 1.66, 95% confidence interval [CI]: 1.27–2.17), initiation of insulin treatment (OR = 1.84, 95% CI: 1.15–2.93), and macrosomia (OR = 1.62, 95% CI: 1.03–2.52).^[8]^ Another national population-based cohort study of 3667,461 singleton deliveries, of which 3108 occurred in women with SCZ, revealed that women with SCZ are more likely to develop diabetic pregnancies (adjusted OR = 1..41 [95% CI: 1.31–1.51]) compared with matched controls.^[9]^ One large-scale meta-analysis including 43,611 deliveries of women with SCZ and 40,948,272 controls found that women with SCZ had higher OR of GDM (2.35, 95% CI: 1.57–3.52).^[10]^ Another meta-analytic review of 2 prospective population-based studies, involving 237 schizophrenic pregnant subjects and 1909 normal pregnant subjects, has demonstrated a 7.76 OR of developing GDM among SCZ pregnant women by reference to their counterparts.^[11]^

Besides these epidemiological links, substantial data suggests an iatrogenic effect. A meta-analysis of 10 studies that included 6213 patients in the antipsychotic-exposed group, 6836 in the antipsychotic-ceased control group, and 1677,087 in the healthy control group, reported an increased risk of GDM in women who use antipsychotics during pregnancy.^[12]^ A retrospective study involving 539 pregnant women with mental disorders including SCZ, showed a significantly greater likelihood of developing GDM in participants who had higher psychotic disease severity or were using specific antipsychotic agents including risperidone and high-dose quetiapine.^[13]^ Similar results were reported by Wang et al (6642 exposed and 1860,290 unexposed pregnancies),^[14]^ and Heinonen et al (1307,487 singleton births),^[15]^ whereas Lin et al, found no increased odds of GDM following antipsychotics intake in early pregnancy.^[16]^

### 3.2. The susceptibility to SCZ-related disorders in offspring issued of diabetic pregnancy

Few human studies have explored the link between prenatal exposure to maternal diabetes and susceptibility to SCZ. Yamasaki et al conducted a study involving 4478 ten-year-old adolescents, to explore any significant association between maternal GDM and self-reported SCZ-characterizing symptoms, notably hallucinations. Their findings showed a greater predisposition to auditory (OR: 4.33) and visual hallucinatory experiences (OR: 6.58) in adolescent offspring of GDM mothers.^[17]^ In a large-scale, population-based cohort study involving 2413,335 live births from 1978 to 2016, Nogueira Avelar e Silva et al found that offspring born to mothers with diabetic pregnancies had an increased risk of developing any psychotic disorder in the first 4 decades of life. More specifically, the hazard ratio for developing SCZ was 1.55 (95% CI: 1.15–2.08). The authors used the 10th International Classification of Diseases (ICD-10) as a reference, with SCZ and related disorders coded as F20-F29.^[18]^ Other data comprising persons with SCZ (n = 142) and their unaffected first-degree relatives (n = 277) suggest that both macrosomia and low birth weight are associated with SCZ risk.^[19]^ Additionally, several reports support the role of GDM in promoting other psychotic disorders in offspring. In the study by Nogueira Avelar e Silva, offspring born to mothers with GDM were more susceptible to anxiety disorders, intellectual disabilities, developmental disorders, and behavioral disorders compared to offspring born to mothers without GDM.^[18]^ Two systematic reviews and meta-analyses over 1256,150 and 784,056 subjects, respectively,^[20,21]^ and 1 large multiethnic clinical longitudinal cohort over 322,323 singleton pregnancies^[22]^ have demonstrated an increased risk of autism spectrum disorder in offspring following maternal diabetic pregnancies. Similar observations were found in a prospective national cohort study in the US, including 66,445 pregnancies and 793 autistic children.^[23]^ Other data suggest an association between maternal diabetes and the risk of attention-deficit/hyperactivity disorder in childhood.^[24]^ This accumulated evidence strongly supports the role of GDM and maternal diabetes in increasing the risk of SCZ and other mental and psychiatric disorders. These findings underscore the relevance of targeted early interventions in the offspring of diabetic pregnancies, highlighting a key area for future research and public health.

## 4. Exploring mechanistic links between gestational diabetes and SCZ

### 4.1. Overview on GDM pathophysiology

The pathophysiology of GDM is complex and not fully understood. During pregnancy, β-cells undergo chronic hyperplasia to compensate for the increased glucose demand of the growing fetus, leading to hyperinsulinemia. GDM is believed to result from the failure of β-cells to adapt to this high glucose demand, leading to their apoptosis, against a backdrop of insulin resistance mediated by gestational hormones, likely in genetically predisposed individuals.^[25]^ β-cell failure may stem from various genetic deficiencies, such as in the potassium voltage-gated channel, KQT-like superfamily, member 1,^[26,27]^ glucokinase,^[28]^ prolactin receptor,^[29]^ and forkhead box protein M1.^[30]^ Both prolactin receptor and forkhead box protein M1, crucial for β-cell expansion and adaptation during pregnancy, may impair the ability of cells to sense plasma glucose or to properly produce and secrete insulin. Inadequate insulin secretion leads to decreased insulin-dependent glucose uptake in skeletal muscle cells, causing hyperglycemia, which overburdens β-cells through an effect called “glucotoxicity.”^[31]^ Insulin resistance arises when the intracellular signals triggered by insulin fail to induce the expression of glucose transporter-4 on the cell membrane, thereby impeding glucose entry into cells.^[25]^ In GDM, insulin resistance is associated with either decreased tyrosine or increased serine/threonine phosphorylation of the insulin receptor, along with diminished expression of insulin receptor substrate-1, phosphatidylinositol 3-kinase, and glucose transporter-4.^[32]^ These post-binding insulin defects in GDM are partly attributed to human placental lactogen hormone, along with placental growth hormone, progesterone, cortisol, and prolactin. Human placental lactogen hormone, a gestational peptide, antagonizes insulin by binding with high affinity to prolactin receptors, facilitating increased maternal glucose delivery to the fetal circulation.^[33]^ Genetic susceptibility may also contribute to insulin resistance, as single nucleotide polymorphisms in the CDK5 regulatory subunit-associated protein 1-like 1 gene, which enhances proinsulin translation in pancreatic βcells, were related to increased risk of GDM.^[34,35]^ Moreover, recent studies have identified newly involved candidates in promoting insulin resistance such as tumor necrosis factor-alpha, which affects insulin signaling by increasing serine phosphorylation of insulin receptor substrate-1 and downregulating tyrosine kinase activity on insulin receptors.^[36,37]^

### 4.2. Current understanding of SCZ etiopathogenesis

Similar to GDM, the pathophysiology of SCZ remains incompletely understood, with the existing data being limited and heterogeneous. SCZ-related abnormalities are believed to result from dysregulation of the dopamine system and its interaction with other neurotransmitter (e.g., glutamate, GABA) systems.^[38]^ Uncovering the cause of these abnormalities is the hope of undergoing research on SCZ.^[39]^ According to the neurodevelopmental model – the predominant theory, SCZ is caused by early-life pathological processes that disrupt brain maturation, starting in utero during the first or early second trimester. These anomalies are triggered by complex interactions between intrinsic (e.g., genetic susceptibility) and extrinsic (e.g., infection and environmental pollutants) factors.^[6,40]^ Regardless of whether the predisposing factors are intrinsic or extrinsic, they are likely to occur at an early stage of life (typically prenatally or perinatally), leading to subclinical alterations that manifest clinically under the influence of additional factors during childhood or adolescence. This is known as the “Two-Hit Hypothesis,” when the early aggressor(s) represent the first hit, and the subsequent factor(s) constitute the second and necessary hit to complete the etiopathogenesis of SCZ.^[41]^

Patients with SCZ display particular genetic polymorphisms. Epidemiological studies suggest that SCZ is a highly heritable component, with heritability estimates of approximately 80%.^[42]^ Various genes have been associated with SCZ, such as disrupted-in-SCZ 1, neuregulin, catechol-o-methyltransferase, dystrobrevin-binding protein 1, regulator of G-protein signaling 4, 5HT2A, and dopamine D3 receptor. However, results regarding these genetic associations remain inconclusive.^[43,44]^

Other factors are also linked to the pathogenesis of SCZ. Among these are prenatal infections involving pathogens, such as influenza virus, cytomegalovirus, herpes simplex virus, Epstein–Barr virus, mycoplasma, *Chlamydia*, and *Toxoplasma gondii*, along with their associated inflammation.^[45]^ Autoimmunity also appears to play a considerable role in the development of SCZ. Increasing evidence suggests that SCZ may be related to a shift in the host immune response toward CNS antigens. This theory is based on the association of SCZ with several autoimmune diseases, suggesting shared etiopathological mechanisms.^[46–50]^ Additionally, individuals with SCZ often exhibit elevated blood levels of pro-inflammatory cytokines^[51,52]^ and autoreactive antibodies against brain antigens.^[53–56]^ Notably, certain antibodies, such as those targeting the n-methyl-d-aspartate-receptor and anti-neural cell adhesion molecule, can induce SCZ-related features.^[54,57]^ Another etiopathogenetic pathway involves dysbiosis, evidenced in individuals with SCZ.^[58]^ Analysis of the human microbiome showed that, at the phylum level, *Proteobacteria* were relatively decreased in SCZ subjects compared to healthy controls. At the genus level, the genus *Anaerococcus* (nonparametric Kruskal–Wallis H score [H] = 8.32) was relatively increased, while *Haemophilus* (H = −11.3), *Sutterella* (H = −12.0), and *Clostridium* (H = −15.9) were decreased in SCZ compared to controls.^[59]^ Finally, SCZ risk is also associated with exposure to environmental pollutants such as NO_2_ and NO_x_ during childhood.^[60,61]^

### 4.3. Mechanistic interplay between GDM and SCZ

**Figure 1.**
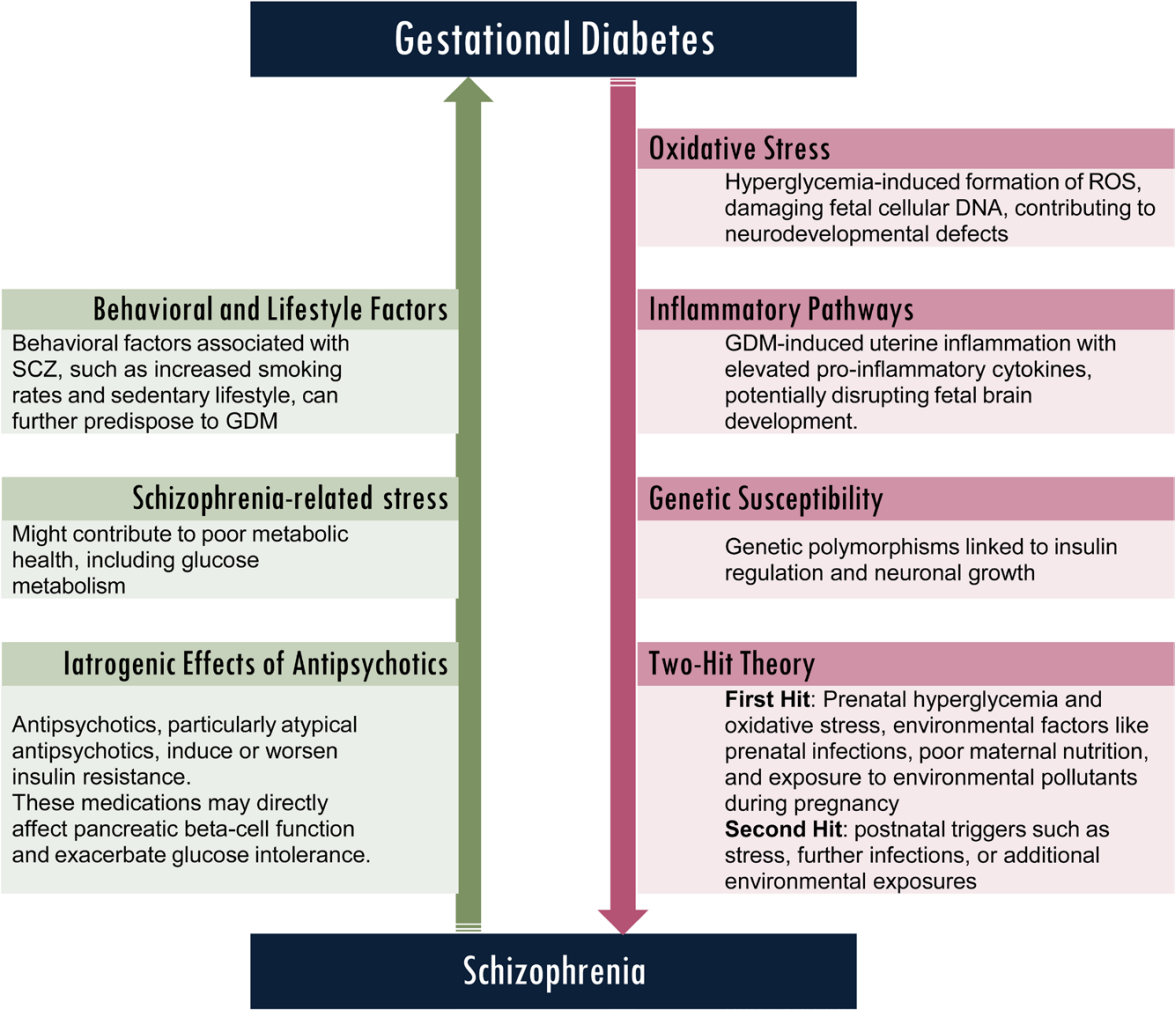
Mechanistic relations and theories linking gestational diabetes mellitus and schizophrenia. DNA = deoxyribonucleic acid, GDM = gestational diabetes mellitus, ROS = reactive oxygen species, SCZ = schizophrenia.

The mechanisms explaining the dual relationship between GDM and SCZ are summarized in Figure 1.

#### 4.3.1. How exposure to hyperglycemia in utero promotes the development of SCZ?

Overall, exposure to hyperglycemia during fetal life may result in neurodevelopmental anomalies and sequelae, potentially increasing susceptibility to SCZ later in life. This exposure acts as the “first hit,” creating a predisposition to develop SCZ later in life. This is followed by a latency period, during which a “second hit” – any SCZ-promoting brain aggressor can trigger progression to the symptomatic stage.^[5]^ At the organ level, gestational hyperglycemia can affect the hippocampus,^[62–65]^ amygdala,^[66]^ thalamus,^[67]^ and hypothalamus of the developing fetal brain,^[68]^ as well as the cerebral cortex.^[69]^ All these brain structures are altered to various degrees in patients with SCZ and are therefore involved in the disease pathogenesis and etiopathogenesis.^[70–73]^

At the molecular level, gestational hyperglycemia is suggested to predispose to SCZ through 3 primary mechanisms in the CNS: hypoxia, oxidative stress, and heightened inflammation.^[5]^ Intrauterine exposure to hyperglycemia favors excessive formation of reactive oxygen species, which can damage the DNA of all fetal cells, including the CNS. This can result in long-lasting neurodevelopmental defects with the subsequent development of psychiatric disorders. In vitro hyperglycemia-induced oxidative stress-mediated damage to the neural tube and brain development.^[74]^ Increased reactive oxygen species production and lipid peroxidation during GDM are accompanied by reduced cellular antioxidant activity – notably catalase and superoxide dismutase – which is likely due to these changes.^[69]^ Oxidative stress may lead to fetal tissue hypoxia by activating pathways that impair mitochondria, altering brain development and neurogenesis, and increasing susceptibility to psychiatric disorders, including SCZ, in offspring.^[75]^ Cerebral hypoxia can further result from systemic microcirculation defects seen during GDM.^[76]^ Furthermore, GDM was shown to induce intrauterine neuroinflammation,^[77]^ which in turn favors SCZ according to the 2-hit hypothesis.^[41]^ This effect is thought to be related to increased activity of pro-inflammatory proteins such as adipocytokines and leptin during hyperglycemic pregnancies.^[77]^

In addition to these 3 mechanisms, excess glucose in fetal circulation can dysregulate the developing brain by disrupting (upregulating or downregulating) glucose-related signaling pathways involved in cognition and behavior. An example of this is brain insulin signaling, which is prone to pathological modifications during GDM.^[78]^ In the hippocampus and cortex, insulin and insulin-like growth factor receptors are particularly more condensed. Additionally, insulin plays a crucial role in the acquisition and maintenance of hippocampal neuroplasticity by facilitating glutamatergic activity and modulating mesolimbic circuits, which are pivotal for motivated and feeding behaviors.^[79]^ Importantly, fetal insulin sensitivity may be impaired during GDM,^[80]^ potentially hindering insulin-dependent neuroplasticity protection and enhancement, thereby increasing the risk of SCZ.

Notably, a recent transcriptome-wide association study has shown the presence of 139 SCZ-related genes involved in multiple placental functions, especially nutrient-sensing capabilities and trophoblast invasiveness.^[81]^ Considering that the placenta may suffer structural and functional compromise during GDM,^[82,83]^ there exists a potential link between SCZ and placental dysfunction during prenatal life in diabetic pregnancies.

#### 4.3.2. How can SCZ promote the development of GDM?

During normal pregnancy, maternal tissue sensitivity to insulin progressively decreases, a physiological mechanism aimed at redirecting glucose to fetal circulation.^[32]^ However, exacerbation of this phenomenon may lead to pathological hyperglycemia. Atypical antipsychotics are usually considered the first-line agents for the management of patients with SCZ. Short-term intake of atypical antipsychotics (olanzapine and aripiprazole) was shown to induce insulin resistance in previously healthy subjects. Particularly, olanzapine can lead to rapid and significant changes in postprandial metabolism, manifesting as a reduction in insulin sensitivity and hyperinsulinemia. This effect was independent of hunger or food intake, indicating a direct iatrogenic effect on insulin sensitivity. This effect may result from olanzapine-induced antagonism of muscarinic receptors (M3) in the pancreatic islet cells, ultimately attenuating insulin release.^[84,85]^ Atypical antipsychotics can also alter insulin response to glycemia changes by antagonizing the serotonergic (5HT(2)) and dopamine D(2)-like receptor-mediated release of insulin.^[86]^ Due to these potential disrupting effects on insulin sensitivity and glucose regulation, atypical antipsychotics may favor the development of GDM.

Additionally, during SCZ, the levels of stress hormones (i.e., catecholamines, cortisol, glucagon) increase likely due to hypothalamic-pituitary-adrenal axis anomalies, which may induce hormonal overproduction or due to medication effects.^[87–89]^ This may favor GDM as the stress-induced elevation in the plasma levels of epinephrine, noradrenaline, glucagon, and cortisol was shown to be correlated with the development of insulin resistance in pregnant women, and was therefore suggested to contribute to the pathogenesis of GDM.^[90]^ Even antipsychotics-naïve patients have genetic features suggestive of central insulin dysregulation, likely contributing to dysglycemia susceptibility.^[91]^ As previously explained, genome analysis suggests that SCZ subjects may be at risk of placental dysfunction. The latter is believed to play a major role in the pathogenesis of GDM and its related adverse effects on maternal health during the gestational state.^[92]^

Lifestyle and behavioral factors may also contribute to GDM development in individuals with SCZ. One notable observation is the greater cardiovascular risk factors among individuals with SCZ with reference to those without SCZ.^[93]^ For example, some data showed that 62% of individuals with SCZ were shown to be current smokers, and have no significant motivation to quit.^[94]^ More specifically, women with SCZ have a 2.40-fold likelihood of smoking compared to non-SCZ counterparts.^[95]^ They also have a significantly higher likelihood of smoking at the beginning and during the first trimester of pregnancy, compared to non-schizophrenic controls.^[8]^ Another cardiovascular risk factor is obesity and overweight, which is significantly more frequent among SCZ women, both before and during pregnancy,^[8,9]^ probably attributed to unhealthy diet and sedentary lifestyle.^[96]^ Furthermore, due to the effects of SCZ on gestational and social life, women with SCZ tend to be older during pregnancy compared to their non-SCZ counterparts.^[9]^ Smoking, overweight/obesity, and advanced maternal age are well-established risk factors for GDM and are all known to increase the risk of having diabetic pregnancies.^[97–99]^

#### 4.3.3. How antipsychotics affect glucose metabolism

Antipsychotic medications, used in treating SCZ, are known for their extensive side effect profiles. These effects range from relatively minor issues such as mild sedation to more severe complications like akathisia, sexual dysfunction, acute dystonia, life-threatening myocarditis, and agranulocytosis. Pertinently, the side effects of antipsychotics also include disruptions in glucose metabolism, a focus of our study.^[100]^

Research has shown that both haloperidol, a first-generation antipsychotic, and olanzapine, a second-generation antipsychotic, impair central glucose sensing in the hypothalamus. This impairment occurs through the inhibition of glucose-induced suppression of endogenous glucose production, mediated by blockages in the vascular endothelial growth factor pathway, the phosphatidylinositol 3-kinase pathway, and kinases that activate K_ATP channels in the hypothalamus.^[101]^ In a study involving 14 healthy, normal-weight men treated with olanzapine (10 mg/d) or haloperidol (3 mg/d) over 8 days, it was observed that olanzapine hindered insulin-mediated glucose disposal and dulled the insulin-induced decline of plasma free fatty acid and triglyceride concentrations. However, neither drug affected endogenous glucose production or the glycerol rate of appearance.^[102]^ Furthermore, Teff et al (2013) administered olanzapine, aripiprazole, or placebo for 9 days to healthy subjects (n = 10 for each group) and noted that while aripiprazole induced insulin resistance, olanzapine led to significant increases in postprandial insulin, glucagon-like peptide 1, and glucagon levels, coinciding with insulin resistance.^[84]^

A nationwide Danish study involving 29,955 schizophrenic patients demonstrated a significant association between exposure to second-generation antipsychotics and diabetic ketoacidosis, caused by insulin deficiency (OR = 2.60; 95% CI: 1.06–6.38), and type 2 diabetes (OR = 1.64; 95% CI: 1.48–1.83).^[103]^ In another cohort, olanzapine and aripiprazole were found to nearly double the incidence of type 2 diabetes in patients with SCZ (adjusted hazard ratio, 1.88; 95% CI: 1.36–2.59 and 2.35; 95% CI: 1.70–3.26, respectively), while clozapine increased the rate fourfold (adjusted hazard ratio, 3.98; 95% CI: 2.77–5.73).^[104]^

## 5. Clinical implications and limitations

Healthcare professionals should be aware of the bidirectional relationship between GDM and SCZ. This finding implies the necessity of considering the risk of SCZ in individuals born with diabetic pregnancies. These individuals may benefit from early interventions for at-risk SCZ. Such interventions may aim to improve suboptimal maturation of neuronal pathways (e.g., via supplementation with pro-cognitive components such as choline, anti-oxidants, and omega-3 type polyunsaturated fatty acids), reduce environmental insults (through social skills training to prevent bullying and social exclusion, and early interventions to prevent drug abuse), and improve resilience with exercise training and cognitive remediation. The increased risk of GDM with SCZ episodes and/or antipsychotic exposure in pregnant women indicates that this group should benefit from close pregnancy monitoring, early testing for GDM, targeting modifiable risk factors, and lifestyle modifications.^[12]^ These challenges can be addressed through expert consensus to provide guidance for clinical practice. Collaboration between psychiatrists and obstetricians is crucial to mitigate poor health outcomes in women with SCZ who are pregnant or wish to become pregnant as well as to ensure the well-being of their children. This interdisciplinary approach would facilitate the development of tailored healthcare strategies, focusing on both maternal and child health, and offer comprehensive support throughout the pregnancy and postpartum period.

Finally, it is important to acknowledge the limitations of existing literature, indicating the need for further research to confirm the interplay between GDM and SCZ. Moreover, to date, it remains unclear whether diabetic pregnancy increases the risk of SCZ or any psychiatric disorders related to prenatal anomalies of neurodevelopment.

Psychoneuroendocrinology, with its need for extensive biological investigations, struggles to establish causal links between neuropsychiatric disorders, such as SCZ, and endocrine/obstetric conditions, such as GDM, due to its complexity. It remains highly speculative to attribute the in-utero brain insults related to maternal GDM as potential promoters of SCZ, as the majority of patients with SCZ have no apparent physical anomalies that could suggest sequelae of early-life aggressors.^[105]^ However, advances in imaging techniques might provide more ultrasensitive detection of CNS changes in individuals with SCZ, ultimately supporting the developmental/2-hit hypotheses, in which GDM and other gestational disorders with similar neuropsychiatric consequences could be a real etiological element. On the other hand, the presence of several confounding factors (mainly those related to lifestyle) during SCZ suggests that GDM risk could be mainly due to these factors rather than due to SCZ itself and that the latter could be, in an unspecific manner, risk factors containing terrain. More specifically, the notion of diabetogenic side effects of anti-SCZ drugs has poor clinical support and requires further confirmation. Nevertheless, apart from iatrogenicity, the tremendous level of psychosocial stress a woman with SCZ can experience, both qualitatively and quantitatively, probably disturbs metabolic and endocrine homeostasis during pregnancy. However, the extent of these disturbances remains unknown and needs to be explored. Thus, data on the reciprocal influence of GDM and SCZ remain incomplete and inconsistent. Therefore, it is important to continue investigating this issue in preclinical studies, as well as large-scale longitudinal and interventional studies.

## 6. Conclusion

Existing evidence suggests that the bidirectional relationship between GDM and SCZ has interesting clinical and mechanistic relevance. GDM may exert oxidative, hypoxic, inflammatory, and metabolic teratogenic effects that can irreversibly alter the early stages of neurodevelopment in the fetus, which induces prenatal vulnerability to SCZ. In contrast, anti-SCZ drugs may aggravate insulin intolerance during pregnancy, thereby shifting the gestational physiological balance toward a hyperglycemic state that may progress to GDM. Moreover, the unhealthy lifestyle of women with SCZ may also contribute to gestational complications, including GDM. In clinical practice, children with a history of maternal GDM before birth may be considered as part of the SCZ risk groups and may be candidates for preventive interventions. Parallel to this, the glycemic levels of schizophrenic women taking antipsychotics may need to be closely monitored during the pre-pregnancy and pregnancy periods.

## Acknowledgments

The author acknowledges Dr Mohamed Amine Haireche for his valuable advice in editing the review. He gives permission to be named.

## Author contributions

**Conceptualization:** Kholoud A. Ghamri.

**Data curation:** Kholoud A. Ghamri.

**Formal analysis:** Kholoud A. Ghamri.

**Investigation:** Kholoud A. Ghamri.

**Methodology:** Kholoud A. Ghamri.

**Project administration:** Kholoud A. Ghamri.

**Resources:** Kholoud A. Ghamri.

**Writing – original draft:** Kholoud A. Ghamri.

**Writing – review & editing:** Kholoud A. Ghamri.
